# Beneficial Effect of Tempol, a Membrane-Permeable Radical Scavenger, on Inflammation and Osteoarthritis in In Vitro Models

**DOI:** 10.3390/biom11030352

**Published:** 2021-02-25

**Authors:** Giovanna Calabrese, Alessio Ardizzone, Michela Campolo, Sabrina Conoci, Emanuela Esposito, Irene Paterniti

**Affiliations:** Department of Chemical, Biological, Pharmaceutical and Environmental Sciences, University of Messina, Viale Ferdinando Stagno D׳Alcontres, 31-98166 Messina, Italy; gcalabrese@unime.it (G.C.); aleardizzone@unime.it (A.A.); campolom@unime.it (M.C.); sabrina.conoci@unime.it (S.C.); ipaterniti@unime.it (I.P.)

**Keywords:** osteoarthritis, inflammation, cartilage degeneration, synthetic molecules, tempol

## Abstract

Osteoarthritis (OA) is one of the most common and widespread diseases which is highly disabling for humans. This makes OA a chronic disease for which it is urgent to find new therapeutic strategies. The inflammatory state in OA contributes to its progression through multiple mechanisms involving the recruitment of phagocytes and leukocytes, inflammatory response, and reactive oxygen species (ROS) production. Tempol (4-hydroxy-2,2,6,6-tetramethylpiperidine-1-oxyl) is classifiable as a piperidine nitroxide, with excellent antioxidant effects, while its anti-inflammatory role is not yet clear. On this basis, we explored its promising biological properties in two in vitro model:, macrophage (J774) and chondrocyte (CC) cell lines. With this aim in mind, we induced inflammation in J774 and CC using lipopolysaccharide (LPS) and Interleukin1β (IL-1β), and after 24, 72 and 168 h of tempol treatment analyzed their effects on cytotoxicity and anti-inflammatory activity. Our data suggested that tempol treatment is able to reduce inflammation and nitrite production in LPS-induced J774 as well as reducing the production of proinflammatory mediators including cytokines, enzymes, and metalloproteases (MMPs) in IL-1β-stimulated CC. Thus, since inflammation and oxidative stress have a crucial role in the pathogenesis and progression of OA, tempol could be considered as a new therapeutic approach for this pathology.

## 1. Introduction

Osteoarthritis (OA) is a widespread chronic disease that causes damage to the cartilage and surrounding tissues [1] and can affect one or more anatomical districts, mainly the knee and hip as well as the spine, ankle, shoulder, and fingers. In 2019, 303 million people were affected by OA [2]. 

OA can be classified as primary or secondary [3]. Primary or idiopathic OA forms occur in previously intact joints without any inciting agent. Aging plays an integral part in this form of OA, as the wear and tear on the joints causes damage to the cartilage, leading to an abnormal repair mechanism. Moreover, water content increases in the superficial and middle zones, resulting from the loss of collagen integrity [4].

The secondary form of OA is caused by an underlying predisposing factor; among these, obesity and joint injury are two of the strongest modifiable risk factors [3]. In particular, rheumatoid arthritis and other inflammatory conditions of the joints lead to joint damage and cartilage breakdown. 

OA develops via a combination of biochemical, cellular, and mechanical processes. It is thought to start from the breakdown, by proteolysis, of the cartilage matrix. The weak matrix is prone to fibrillation and erosion, and results in the release of proteoglycans and collagen fragments into the synovial fluid. This process induces an inflammatory response in the synovium, which causes further cartilage degradation. As the cartilage becomes weak it begins to thin out, causing the joint space to narrow

Although the disease has been well studied, its etiology is not fully known. A multifactorial etiopathogenesis is supposed, which could include a genetic, traumatic, or mechanical components [5]. Currently, there is no definitive treatment for the pathology; the current OA drugs are mainly used to relieve the symptoms like severe pain and inflammation, while “nonpharmacological” forms of therapy such as physiotherapy, thermotherapy, and electrostimulation are becoming commonplace [6,7,8]. Therefore, given the chronicity of the disease and the lack of a definitive cure, it is certainly useful to analyze and test new drugs that could be useful both in the management of musculoskeletal pain and in counteracting inflammation due to joint degeneration. Many articles have shown how the modulation of the inflammatory pathways could be an effective strategy for the treatment of OA [9], thus highlighting the role of conventional inflammatory factors in the progression of OA. 

Moreover, the overproduction of ROS and the induction of oxidative stress in chondrocytes are some of the major contributors to OA pathogenesis [10,11,12,13]. Many studies have shown that the ROS levels are highly upregulated in the human OA cartilage and CC [12,14,15,16]. It has been shown, in an in vitro study, that stimulation of human OA CC and mouse CC with IL-1β increases the production of the cellular and mitochondrial ROS that promotes inflammation and apoptosis [17,18] showing that oxidative stress positively correlates with collagen degradation [12] suggesting a role of ROS in cartilage matrix catabolism. Thus, since oxidative stress and inflammation have detrimental effect on joint health and function, targeting these pathways might be of therapeutic importance for the management of OA. In recent years, the effects of tempol, a synthetic molecule with powerful biological properties, have been investigated and deepened [19,20,21]. 

Specifically, tempol (4-hydroxy-2,2,6,6-tetramethylpiperidine-1-oxyl) is classifiable as a piperidine nitroxide having excellent antioxidant effects with nontoxic activity [22]. This antioxidant function is related to the ability of nitroxide radicals to scavenge free radicals and oxidize transition metals in the reduced state, as well as catalyzing the disproportionation of superoxide, facilitating hydrogen peroxide metabolism, and inhibiting Fenton chemistry [23]. Furthermore, this class of compounds has been recognized as having superoxide dismutase (SOD)-like activity, given their ability to decrease superoxide ion levels and modulate nitric oxide (NO) levels [24,25]. In addition to all these properties, tempol also has the ability to penetrate the cell membrane [26] (unlike other antioxidant species that have no intracellular activity) and therefore to increase the antioxidant power. Hence, on this basis, the purpose of our study was to evaluate the anti-inflammatory and antiosteoarthritic tempol properties in both an inflammatory and an OA model in vitro. 

## 2. Materials and Methods 

### 2.1. Cell Cultures

In this study we used two cell lines, murine macrophage cell line (J774) and human chondrocyte cell line (CC) (C20A4 -Sigma-Aldrich, Milan, Italy). J774 cells were maintained in RPMI-1640 culture medium supplemented with 2 mM L-Glutamine, antibiotic–antimycotic solution (Pen/Strep/Amphotericin, Sigma-Aldrich Company Ltd, Milan, Italy), 10% fetal bovine serum (FBS), at 37 °C and 5% CO_2_ in a humidified atmosphere. The cells were maintained in culture until 80% confluence and the medium replaced twice a week. CC were grown in complete CC culture medium and maintained in humidified environment at 37 °C and 5% CO_2_/95% air atmosphere. The medium was replaced twice a week and cells were cultured until confluence was reached.

### 2.2. Cytotoxicity Assay 

The cytotoxicity of tempol on J774 and CC was determined using MTT [3-(4,5-dimethylthiazol-2-yl)-2,5-diphenyltetrazolium bromide] colorimetric assay as reported previously [27]. Briefly, J774 (5 × 10^3^ cells/well) were cultured in 96 wells and after 24 h fresh medium containing several concentrations of tempol (0.1, 0.2, 0.5, 1 mM dissolved in 1% of DMSO) were added and incubated with LPS (1 µg/mL, of *Escherichia coli* serotype, 055:B5; Sigma-Aldrich, Milan, Italy). After 24 h the cells were washed and 200 µL of MTT solution (1 mg/mL in FBS-free medium) was added to each well and incubated for 2 h. Following 2 h incubation, the medium was removed, each well washed 2 times using cold phosphate-buffered saline (PBS), and the formed crystals melted using 200 μL of DMSO. The optical density of the formazan product in solution was then measured with a microplate reader at 570 nm. In another set of experiments CC (5 × 10^3^ cells/well) were cultured in 96 wells and after 24 h fresh medium containing tempol at two different concentrations (0.5 mM, 1 mM) was added and incubated with IL-1β (10 ng/mL). After 24 h the cells were washed and 200 µL of MTT solution was added to each well and the MTT assay was carried out as previously described.

### 2.3. Griess Nitrite Assay 

For Griess nitrite (NO^-^_2_) assay, J774 cells were cultured in medium containing tempol (0.1, 0.2, 0.5, 1 mM) for 24, 72 and 168 h, washed with fresh medium and treated with 1 µg/mL of LPS. After 24 h, 100 µl of the culture medium was mixed with an equal volume of Griess reagent and incubated at room temperature for 10 min according to the manufacturer’s instructions. The absorbance at 540 nm was recorded using a microplate reader [28]. 

### 2.4. MMP1 and MMP3 Activity Evaluation

For MMPs activity evaluation, supernatants from CC treated with tempol and stimulated with IL-1β (10 ng/mL) after 24 h were collected and the levels of MMP1 and MMP3 measured by ELISA (Invitrogen, ThermoFisher Scientific, Waltham, MA, USA) according to the manufacturer’s instructions.

### 2.5. ROS Assay

To measure intracellular reactive oxygen species, we used the Cellular ROS/Superoxide detection assay kit (Abcam, ab139476, Cambridge, MA, USA) [29]. CC cells plated in a black 96-well plate (20,000 cells per well) were incubated with ROS/Superoxide detection mix (2 μM) for 30 min at 37 °C in darkness. As positive and negative controls, the ROS inducer pyocyanin (200 μM) and ROS inhibitor N-acetyl-L-cysteine (5 mM) were used 1 h or 30 min in advance, respectively. Cells were then washed twice with washing buffer and immediately observed for fluorescein and rhodamine in a Molecular Devices Spectramax i3x. Cellular protein content was determined with a BCA-protein kit from Pierce (Thermo Fisher Scientific, Waltham, MA, USA), and the data are presented as ROS normalized to protein.

### 2.6. Determination of Malondialdehyde (MDA) Levels

CC cells (1 × 10^5^ cells/well) were seeded in poly-L-lysine-coated six-well plates. The cells were harvested to detect the levels of malondialdehyde (MDA) using the MDA assay kit as previously described [30].

### 2.7. Quantitative Realtime PCR (qRT-PCR)

For qRT-PCR analyses total RNA from CC, treated with tempol and stimulated with IL-1β (10 ng/mL), was isolated using RNeasy Mini Isolation Kit (Qiagen, Germantown, MD, USA) and quantified as previously described [31]. cDNA was synthesized from 1μg of total RNA using M-MLV Reverse Transcriptase (Life Technologies, Monza, Italy). qRT-PCR was performed using SYBR Green method on a 7900HT Real Time PCR (Applied Biosystems). qRT-PCR analyses were performed on three independent experiments, each sample was tested in triplicate and gene expression was measured using the 2-ΔΔCt method. RNA from control cells was used as reference for relative expression quantitation. The following specific primers: COX2, IL-1β, IL-6, iNOS, TNF-α, and MMPs (1, 3, 9, 13) were used and designed using primer blast. Results were normalized to the levels of glyceraldehyde 3-phosphate dehydrogenase (GAPDH). Oligonucleotide sequences are reported in the Table 1.

### 2.8. Materials

J774 and CC (C20A4 -human chondrocyte cell line) were purchased from Sigma Aldrich, as well as tempol, MTT, LPS, antibiotic–antimycotic solution (Pen/Strep/Amphotericin, Sigma-Aldrich Company Ltd, Milan, Italy) and FBS. L-Glutamine was purchased from Euroclone. All stock solutions were made in nonpyrogenic saline (0.9 % NaCl, Baxter, Milan, Italy). All other chemicals were of the highest commercial grade available.

### 2.9. Statistical Analysis

Statistical analysis was performed by one-way ANOVA followed by Bonferroni multiple comparisons test where appropriate. Bonferroni method has been used as post hoc test when the ANOVA reported statistically significant differences, to evaluate the differences between the individual times or treatment groups. For all experiments, *p* < 0.05 was considered to be significant.

## 3. Results

### 3.1. Effects of Tempol on Macrophages Cell Viability 

**Figure 1:**
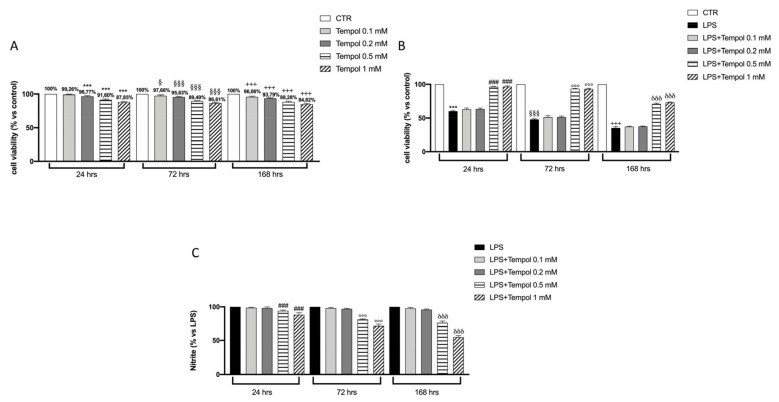
Effects of tempol on J774 cell viability and nitric oxide production. MTT test performed on J774, without and with LPS stimulation, treated with tempol (0.1, 0.2, 0.5 and 1 mM) for 24, 72 and 168 h (**A**, **B**). Griess assay performed on the supernatant of LPS-stimulated J774 treated with different concentrations of tempol for 24, 72 and 168 h (**C**). Control (CTR), untreated J774 cells, LPS, J774 stimulated with LPS. Data are represented as the means ± SD of three independent experiments. One-way ANOVA test. ^§^ compare the LPS group 72 h to CTR group; ^***^ *p* < 0.001 vs. CTR 24 h; ^§§§^ *p* < 0.001 vs. CTR 72 h; ^+++^ *p* < 0.001 vs. CTR 168 h; ^###^ *p* < 0.001 vs. LPS 24 h; ^°°°^ *p* < 0.001 vs. LPS 72 h; ^δδδ^ *p* < 0.001 vs. LPS 168 h.

To evaluate the cytotoxic effect of different concentrations (0.1, 0.2, 0.5, 1 mM) of tempol on both J774 and LPS-induced J774 cells, we performed a MTT colorimetric test. The data reported in Figure 1A show that tempol does not exhibit significant cytotoxic effects on J774 cells at all lower concentrations used (0.1–0.2 mM) at all timepoints of treatment, while after 168 h of incubation it is possible to notice a slight trend of reduction in cell viability, especially at the higher concentration (Figure 1A) and especially at 72 and 168 h. Instead, J774 cells induced with LPS displayed a significantly reduction in cell viability as shown in Figure 1B, whereas treatment with tempol only showed a recovery of cell viability at the highest concentrations of 0.5 and 1 mM compared to treatment with LPS alone (Figure 1B).

### 3.2. Effects of Tempol on LPS-Induced NO Production in J774 Cells

In order to evaluate the anti-inflammatory property of tempol, NO levels of J774 cells stimulated with LPS and treated with the selected concentrations (0.1–1.0 mM) for 24, 72 and 168 h were measured; our results showed slight cytotoxicity, especially at high concentrations of tempol. Our data showed that NO produced by LPS-stimulated J774 cells was significantly inhibited only at the two highest concentrations in time dependent manner (Figure 1C), even if the 1.0 mM concentration was more effective. Since only the two highest concentrations (0.5 and 1.0 mM) of tempol were able to significantly increase cell viability as well as reduced NO levels in LPS-stimulated J774, we decided to perform all further experiments with only these two concentrations.

### 3.3. Effects of te3mpol on CC Cell Viability 

**Figure 2:**
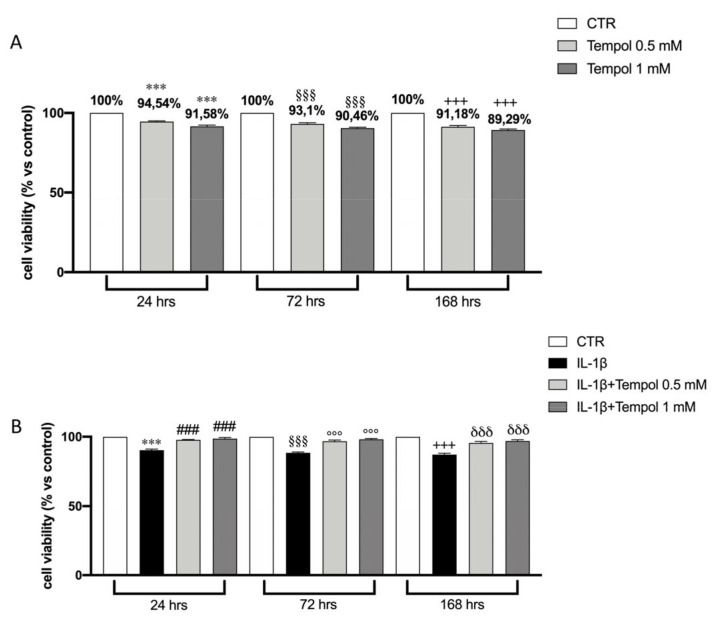
Effects of tempol on CC cell viability. MTT test performed on CC treated with tempol (0.5 and 1 mM) for 24, 72 and 168 h (**A**). MTT test performed on IL-1β-stimulated CC and treated with tempol (0.5 mM and 1.0 mM) for 24, 72 and 168 h (**B**). CTR, untreated CC; IL-1β, Il-1β stimulated CC; Data are represented as the means ± SD of three independent experiments. One-way ANOVA test. ^***^ *p* < 0.001 vs. CTR 24 h; ^§§§^ *p* < 0.001 vs. CTR 72 h; ^+++^ *p* < 0.001 vs. CTR 168 h; ^###^ *p* < 0.001 vs. IL-1β 24 h; ^°°°^ *p* < 0.001 vs. IL-1β 72 h; ^δδδ^ *p* < 0.001 vs. IL-1β 168 h.

Before carrying out further in vitro tests on CC cells, it was necessary to evaluate the cytotoxic effects of tempol at the two selected concentrations (0.5 and 1.0 mM) on CC alone and CC stimulated with IL-1β at 10 ng/mL. The effects of tempol on cell viability are shown in Figure 2A. Our results showed that tempol, at both concentrations, slightly reduces cell viability at all times analyzed (Figure 2A), and IL-1β at 10 ng/mL induces a significant reduction of cell viability at all timepoints (Figure 2B). 

We observed that tempol, at both concentrations, displayed a recovery of cell viability compared to treatment with IL-1β alone (Figure 2B).

### 3.4. Effects of Tempol on IL-1β-Induced Inflammatory me3diators and MMPs 

**Figure 3:**
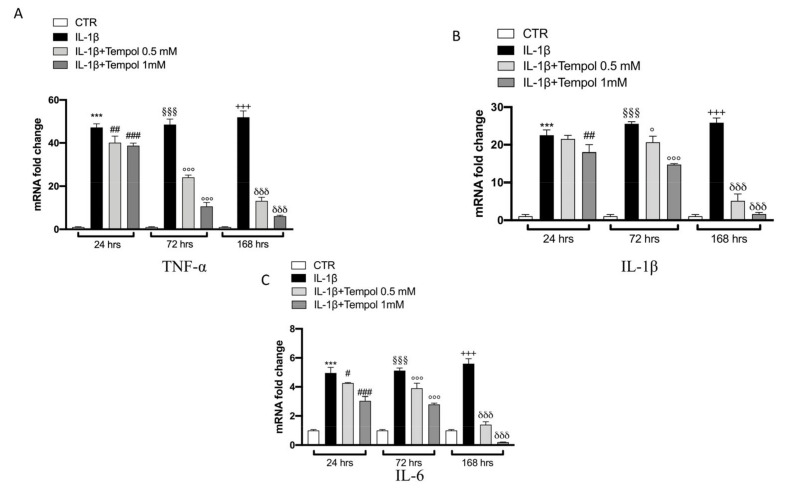
Effects of tempol on IL-1β-induced inflammatory cytokines gene expression. qRT-PCR performed with IL-1β (**B**), IL-6 (**C**) and TNF-α (**A**) on IL-1β-stimulated CC and treated with tempol (0.5 and 1.0 mM) for 24, 72 and 168 h. GAPDH was used as endogenous controls. CTR, untreated CC; IL-1β, IL-1β-stimulated CC; IL-1β+tempol, IL-1β-stimulated CC and treated with tempol 0.5 and 1.0 mM. Data are representative of at least three independent experiments. One-way ANOVA test. ^°^ compare the LPS-tempol group 72 h to IL1 beta group; ^***^ *p* < 0.001 vs. CTR 24 h; ^§§§^ *p* < 0.001 vs. CTR 72 h; ^+++^ *p* < 0.001 vs. CTR 168 h; ^###^ *p* < 0.001 vs. LPS 24 h; ^##^*p* < 0.01 vs. LPS 24 h; ^#^ *p* < 0.05 vs. LPS 24 h; ^°°°^ *p* < 0.001 vs. LPS 72 h; ^δδδ^ *p* < 0.001 vs. LPS 168 h.

**Figure 4:**
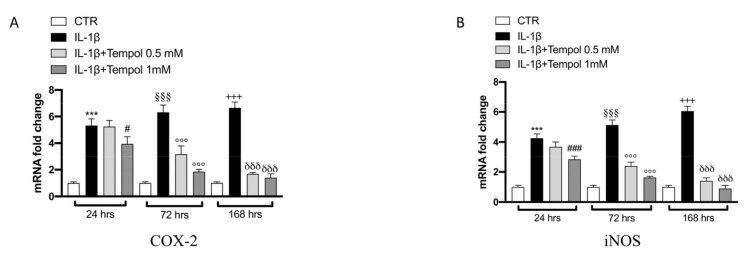
Effects of tempol on IL-1β-induced inflammatory enzymes gene expression. qRT-PCR performed with COX-2 (**A**) and i-NOS (**B**) on IL-1β-stimulated CC and treated with tempol (0.5 and 1.0 mM) for 24, 72 and 168 h. GAPDH was used as endogenous controls. CTR, untreated CC; IL-1β, IL-1β-stimulated CC; IL-1β+tempol, IL-1β-stimulated CC and treated with tempol 0.5 and 1.0 mM. Data are representative of at least three independent experiments. One-way ANOVA test. ^***^ *p* < 0.001 vs. CTR 24 h; ^§§§^ *p* < 0.001 vs. CTR 72 h; ^+++^ *p* < 0.001 vs. CTR 168 h; ^###^ *p* < 0.001 vs. LPS 24 h; ^#^ *p* < 0.05 vs. LPS 24 h; ^°°°^ *p* < 0.001 vs. LPS 72 h; ^δδδ^ *p* < 0.001 vs. LPS 168 h.

**Figure 5:**
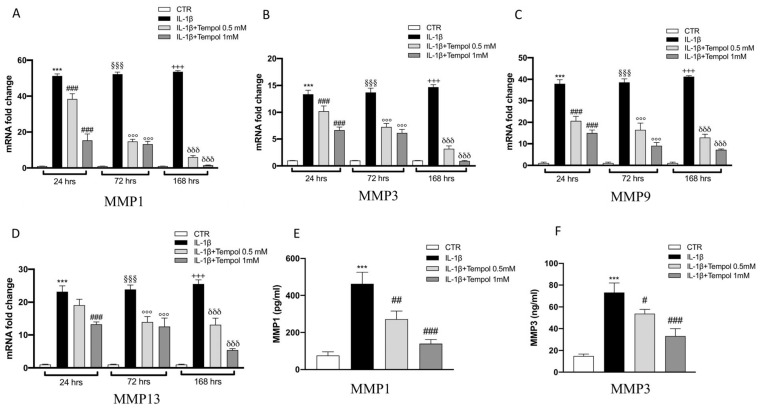
Effects of tempol on IL-1β-induced MMPs activity and gene expression. IL-1β-stimulated CC were treated with 0.5 and 1.0 mM of tempol for 24, 72 and 168 h. Expression of MMP1 (**A**), MMP3 (**B**), MMP9 (**C**) and MMP13 (**D**) was evaluated using real time-PCR. CTR, control untreated CC; IL-1β, IL-1β-stimulated CC; IL-1β+tempol, IL-1β-stimulated CC and treated with tempol 0.5 and 1.0 mM. GAPDH was used as endogenous controls. Moreover, MMP activity was evaluated by ELISA kit assay (**E**,**F**). Data are representative of at least three independent experiments. One-way ANOVA test. ^***^ *p* < 0.001 vs. CTR 24 h; ^§§§^ *p* < 0.001 vs. CTR 72 h; ^+++^ *p* < 0.001 vs. CTR 168 h; ^###^ *p* < 0.001 vs. LPS 24 h; ^##^ *p* < 0.01 vs. LPS 24 h; ^#^ *p* < 0.05 vs. LPS 24 h; ^°°°^ *p* < 0.001 vs. LPS 72 h; ^δδδ^ *p* < 0.001 vs. LPS 168 h.

To evaluate the protective effects of tempol on IL-1β-stimulated CC we performed qRT-PCR to analyze the gene expression of inflammatory mediators (Figure 3 and Figure 4) and MMPs (Figure 5) after 24, 72 and 168 h of treatment. Our data indicate that after 24 h of treatment with tempol at 0.5 mM no significant effect was noticeable in IL-1β (Figure 3B), COX-2 and iNOS (Figure 4A,B respectively) as well as in MMP13 (Figure 5D) expression levels; while a significant reduction was visible for TNF-α and IL-6 (Figure 3A,C respectively) and for MMP1 and MMP13 (Figure 5A–C, respectively) compared to the IL-1β stimulated CC. On the other hand, treatment with tempol 1.0 mM was able to reduce the levels of all analyzed genes in a statistically significant manner, at all timepoints analyzed, compared to the IL-1 β group. In order to assess the effects of tempol on IL-1β-stimulated MMP1 and MMP3 production we performed two ELISA assays. The results showed that MMP1 and MMP3 production in IL-1β-stimulated CC increased significantly (MMP1, 462.74 ± 62.71 pg/mL; MMP3, 73.11 ± 8.81 ng/mL) compared to the control (MMP1, 76.36 ± 19.89 pg/mL; MMP3, 14.88 ± 1.81 ng/mL) and that the MMP1 and MMP3 activities of IL-1β-induced CC were significantly reduced by tempol at both concentrations (Figure 5E,F respectively) (MMP1: tempol 0.5 mM_272.49 ± 43.83 pg/mL and tempol 1.0 mM_139.85 ± 21.80 pg/mL; MMP3: tempol 0.5 mM_ 53.83 ± 3.95 ng/mL, tempol 1.0 mM_33.13 ± 6.81 ng/mL) (Figure 5E,F, respectively). To confirm the anti-inflammatory effect of tempol we also evaluated the levels of proinflammatory cytokines such as TNF-α and IL-1β by Elisa kit assay (see Supplementary Materials Figure S1), as well as which we observed the protein expression of COX-2 and iNOS by Western blot (see Supplementary Materials Figures S2 and S3, respectively). The data obtained confirmed the capacity of tempol to reduce, both at 72 and 168 h, the inflammatory markers. 

### 3.5. Antioxidant Effects of Tempol on Oxidative Stress

**Figure 6:**
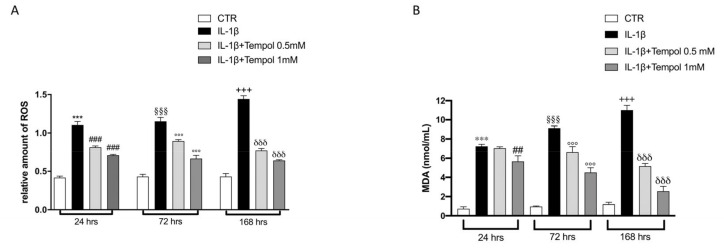
Effects of tempol on oxidative stress. Evaluation of oxidative stress was assessed by ROS assay and MDA assay. CTR, control untreated CC; IL-1β, IL-1β-stimulated CC; IL-1β+tempol, IL-1β-stimulated CC and treated with tempol 0.5 and 1.0 mM. Treatments with tempol 0.5 and 1.0 mM notably reduced intracellular ROS concentrations increment IL-1β-induced (**A**) as well as prevented the elevated lipidic peroxidation caused by IL-1β-stimulation (**B**). Data are representative of at least three independent experiments. One-way ANOVA test. ^***^ *p* < 0.001 vs. CTR 24 h; ^§§§^ *p* < 0.001 vs. CTR 72 h; ^+++^ *p* < 0.001 vs. CTR 168 h; ^###^ *p* < 0.001 vs. LPS 24 h; ^##^ *p* < 0.01 vs. LPS 24 h; ^°°°^ *p* < 0.001 vs. LPS 72 h; ^δδδ^ *p* < 0.001 vs. LPS 168 h.

Another important issue in OA pathogenesis is the production of ROS and induction of oxidative stress in CC. Thus, we evaluated the antioxidant effect of tempol in reducing ROS production by ROS assay. We observed that there was a significant increase of ROS production after IL-1β-stimulation compared to CC control group at each timepoint (Figure 6A); whereas the treatment of tempol significantly decreased ROS production in a concentration dependent manner at each timepoint (Figure 6A). 

ROS production correlates to and determinates the lipidic peroxidation of the membranes that was evaluated by MDA assay. We observed an important increase in the levels of MDA after IL-1β-stimulation compared to CC control group at each timepoints (Figure 6B); treatment with tempol was able to significantly reduce MDA levels in a concentration-dependent manner at each timepoints (Figure 6B). 

## 4. Discussion

Osteoarthritis (OA) is one of the most disabling joint disorders worldwide, still lacking a resolutive therapeutic strategy [2]. Despite the fact that OA is correlated to genetic predisposition and epigenetic regulation [32], it is possible to act by attenuating the inflammatory condition that underlies the pathology [33,34]. The inflammatory state in OA, and in particular the increase in the expression of some inflammatory mediators, contributes to the progression of the disease through multiple mechanisms that involve the recruitment of phagocytes and leukocytes [35,36], intrinsic inflammatory response [35,37] and ROS production [38]. Therefore, the control of the inflammatory condition could represent one of the strategies for better management of the symptoms and the advancement of OA. Multiple studies have evaluated the excellent antioxidant activity of tempol to counteract oxidative stress conditions [39,40] due to the presence of a nitroxide ion, which gives it the ability to be a “radical scavenger”; while its anti-inflammatory role is still under continuous investigation [41]. Based on this knowledge, the aim of this study was to investigate, for the first time, the capacity of tempol to reduce the inflammatory stimuli associated with OA pathogenesis. In particular, for the in vitro model of OA, a CC line was used in order to have a model that is as appropriate as possible and that attempts to mimic the factors and conditions that trigger OA and cause its progression [42], as well as being reliable for verifying new therapeutic potentials. Cell viability tests performed by MTT assay on both J774 and CC cell lines reveal a slight cytotoxicity of tempol, especially at the high concentrations used, compared to the control. As observed from our results, when CC were stimulated with IL-1β they showed a slight reduction in cell viability instead treatment of tempol, especially at 0.5 and 1.0 mM, demonstrated a recovery in cell viability time-dependent manner. Since the presence of macrophages concentrated in the synovial tissue contribute to the promotion of the inflammatory microenvironment and contribute to the onset and progression of OA by interacting with CC and fibroblasts [43,44], in this study we investigated the anti-inflammatory activity of tempol using a macrophage cell line. Specifically, we stimulated J774 cells with LPS for 24 h and observed that the induction of the inflammatory state determinates a reduction in cell viability in a time-dependent manner; instead the treatment with tempol, especially at 0.5 and 1 mM, was able to recover cell viability, as the control, showing a protective activity. Inflammation is a major factor associated with the risk of both progression of cartilage loss and signs and symptoms of OA, including joint pain, swelling, and stiffness, indicators of synovitis [45]. Synovitis, involving the infiltration of mononuclear cells into the synovial membrane and the production of proinflammatory mediators, including IL-1β, TNF-α and chemokines, is common in early stage and late stage disease [42]. Therefore, considering the relevant role of these mediators in the initiation of the inflammatory cascade in OA, in this study we evaluated their expressions by qRT-PCR. Our results showed that tempol, at concentrations of 0.5 and 1 mM, was able to decrease the expression of cytokines, especially of TNF-α. IL-1β and TNF-α increase the synthesis of prostaglandin E2 (PGE2) by stimulating COX-2 gene expression and upregulating the production of NO via inducible nitric oxide synthase iNOS [45,46]. Here we observed that at 0.5 mM, but even more at 1.0 mM, tempol has shown promise effects in decreasing iNOS and COX-2 expressions. Several clinical studies [47,48] have pointed out that in patients with OA there are elevated levels of MMPs; the action of these proteases contributes enormously to cartilage degradation, increasing joint damage, thus accelerating the progression of joint destruction [49]. Since MMPs play an important role in the pathogenesis of OA, an extensive analysis of these proteases and specifically of MMP1, MMP3, MMP9 and MMP13 was performed; treatment with tempol at 1.0 mM considerably reduced their expression, as is clearly demonstrated by our results. Although in the last decade much knowledge about inflammatory mediators in OA has been gained, further studies are needed to better define the mechanisms by which these factors tip the balance between homeostasis and activation to promote matrix destruction and cell death. In line with this idea, our study demonstrates for the first time the important anti-inflammatory properties of tempol in counteracting the response of CC to the inflammatory insults that characterized OA. 

## 5. Conclusions

In conclusion, although the present work needs further validation, especially in vivo, it supports the idea that tempol treatment, at 0.5 and 1.0 mM concentrations, can be considered as a new therapeutic approach for inflammatory states, including OA, because in addition to having a protective effect on cell viability, it is able to modulate NO production in J774 and all inflammatory mediators in CC, even after only 24 h of treatment.

## Figures and Tables

**Table 1:** qRT-PCR oligonucleotide sequences.

Homo Sapiens Gene	Forward	Reverse
**COX2**	TGCAGTGAGCGTCAGGAG	CAAGGATTTGCTGTATGGCT
**IL-1ß**	GAAGTACCTGAGCTCGCCATGGAA	CGTGCAGTTCAGTGATCGTACAGG
**IL-6**	CAAATTCGGTACATCCTC	CTGGCTTGTTCCTCACTA
**iNOS**	TCACCTACCACACCCGAGA	CGCTGGCATTCCGCACAA
**TNF-α**	CAAGCCTGTAGCCCATGTTGT	CCAAAGTAGACCTGCCCAGAC
**MMP1**	CGACTCTAGAAACACAAGAGCAAGA	AAGGTTAGCTTACTGTCACACGCTT
**MMP3**	GAACAATGGACAAAGGATACAACA	TTCTTCAAAAACAGCATCAATCTT
**MMP9**	CACTGTCCACCCCTCAGAGC	GCCACTTGTCGGCGATAAGG
**MMP13**	GTGGTGTGGGAAGTATCATCA	GCATCTGGAGTAACCGTATTG
**GAPDH**	GGAGAAGGCTGGGGCTCAT	TGGGTGGCAGTGATGGCA

## Data Availability

The data presented in this study are available on request from the corresponding author.

## Supplementary Materials

The following are available online at https://www.mdpi.com/2218-273X/11/3/352/s1, **Figure S1**: Effects of tempol on IL-1β and TNF-α expression. ELISA kit was performed both 72 and 168 h after CC stimulation with IL-1β. TNF-α and IL-1β levels were overexpressed on IL-1β-stimulated CC compared to CTR group at both timepoints (A, B, C, D). Treatment with tempol (0.5 mM and 1.0 mM) significantly reduced levels of both cytokines after 72 h and 7 days (A, B, C, D). Data are representative of at least three independent experiments. One-way ANOVA test. *** *p* < 0.001 vs. CTR; ## *p* < 0.01 vs. IL-1β; ### *p* < 0.001 vs. IL-1β. **Figure S2**: Effects of tempol on COX-2 expression. Western blot analysis demonstrated an increase of COX-2 expression after IL-1β stimulation, both at 72 and 168 h, compared to CTR group (A,A1 and B,B1 respectively). Tempol (0.5 mM and 1.0 mM) significantly decreased these levels at both timepoints (A,A1 and B,B1). Data are representative of at least three independent experiments. One-way ANOVA test. *** *p* < 0.001 vs. CTR; ## *p* < 0.01 vs. IL-1β; ### *p* < 0.001 vs. IL-1β. **Figure S3**: Effects of tempol on iNOS expression. Western blot analysis of iNOS showed a significant increase in CC stimulated with IL-1β after 72 and 168 h (A,A1 and B,B1. Treatment with tempol (0.5 mM and 1.0 mM) considerably reduced iNOS expression already after 72 h and even more after 168 h (A,A1 and B,B1). Data are representative of at least three independent experiments. One-way ANOVA test. *** *p* < 0.001 vs. CTR; ### *p* < 0.001 vs. IL-1β.
