# Supplementary material for: Beneficial Effect of Tempol, a Membrane-Permeable Radical Scavenger, on Inflammation and Osteoarthritis in In Vitro Models
Source: Biomolecules. 2021 Feb 25;11(3):352. doi: 10.3390/biom11030352 (PMC7996488; doi:10.3390/biom11030352)
Supplement: Supplementary file 1 [file biomolecules-11-00352-s001.pdf]

biomolecules

MDPI

Methods for Supplementary Materials

ELISA assay for IL-1β and TNF-α

The levels of IL-1β and TNF-α were performed by ELISA kit as previously described. In detail, supernatants were collected, aliquoted, and stored at −20 °C. IL-1β and TNF-α were measured by ELISA kits according to the manufacturer's instructions (Elisa kit MyBiosource).

Western Blot Analysis

Western blot analysis was performed as previously described. CC cells were washed two times with ice-cold phosphate-buffered saline (PBS) harvested and resuspended in Tris-HCl 20 mM pH 7.5, NaF 10 mM, 150 μL NaCl, 1% Nonidet P-40 and protease inhibitor cocktail (Roche). After 40 min., cell lysates were centrifuged at 16000 g for 15 min. at 4 °C. Protein concentration was estimated by the Bio-Rad protein assay using bovine serum albumin as standard. Samples were heated at 95°C for 5 min., and the same amounts of protein separated on 12% SDS-PAGE gel and blotted to a PVDF membrane (Immobilon-P). The membrane was incubated overnight at 4 °C with anti-iNOS (1:500, BDTransduction); anti-COX-2 (1:500, sc-376861 Santa-Cruz Biotechnology, Dallas, Texas, USA). The signals were detected with a chemiluminescence detection system reagent according to the manufacturer's instructions (Super Signal West Pico Chemiluminescent Substrate, Pierce Thermo Scientific, Rockford, IL. USA). Relative expression of bands for iNOS, COX-2 was imported to analysis software (Image Quant TL, v2003); moreover, to ascertain that blots were loaded with equal amounts of protein lysate, they were also incubated with the antibody β-actin (1:500; Santa Cruz Biotechnology). The relative expression of the protein bands was calculated by densitometry with Bio-Rad ChemiDoc™ XRS + software. Molecular weight standards (10–250 kD) were used to define molecular weight positions, and as reference concentrations for each protein, as previously described.

Supplementary Figures

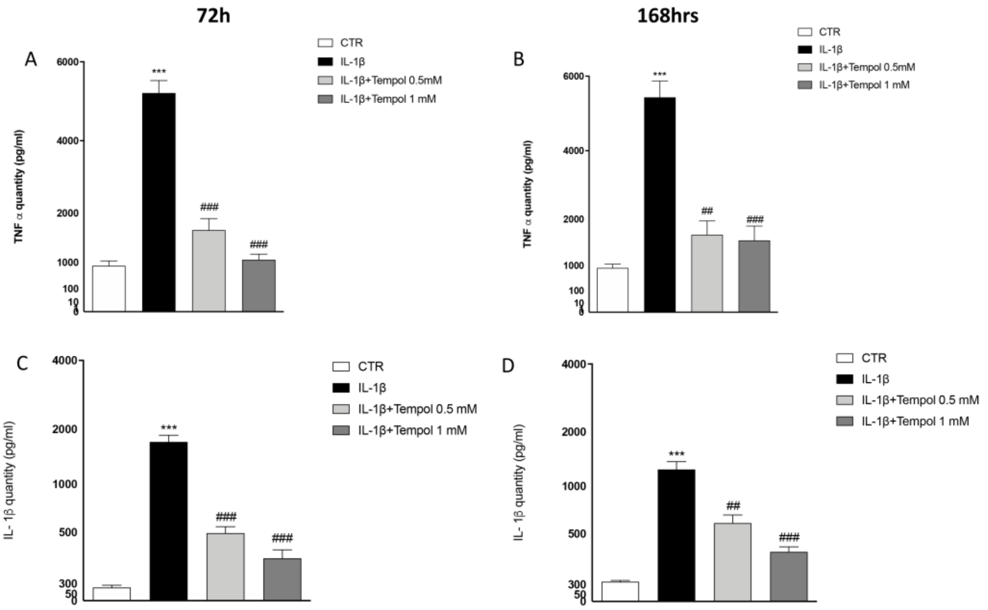

Figure 1S

Figure S1. Effects of tempol on IL-1β and TNF-α expression. ELISA kit was performed both 72 and 168 hrs after CC stimulation with IL-1β. TNF-α and IL-1β levels were overexpressed on IL-1β-stimulated CC compared to CTR group at both timepoints (A, B, C, D). Treatment with tempol (0.5mM and 1.0mM) significantly reduced levels of both cytokines after 72 hrs and 7 days (A, B, C, D). Data are representative of at least three independent experiments. One-way ANOVA test. *** $p < 0.001$ vs. CTR; ## $p < 0.01$ vs. IL-1β; ### $p < 0.001$ vs. IL-1β.

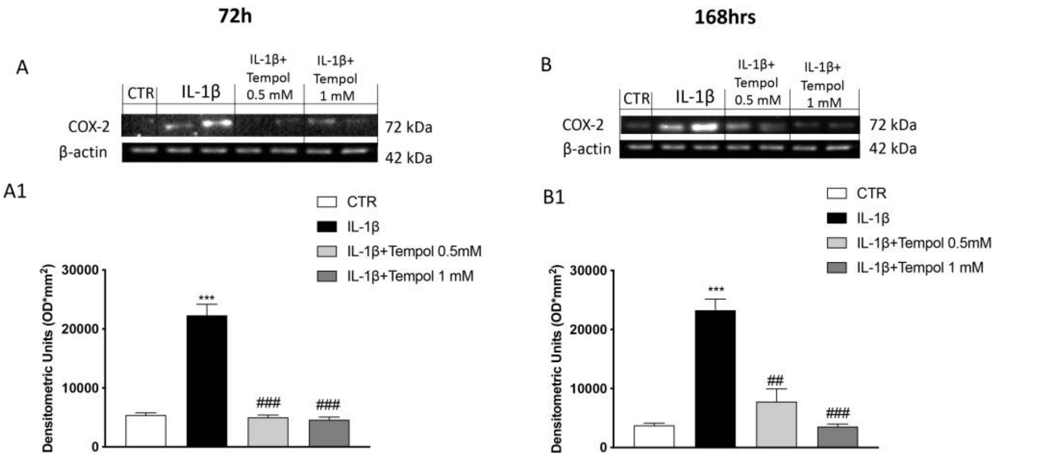

Figure 2S

Figure S2. Effects of tempol on COX-2 expression. Western blot analysis demonstrated an increase of COX-2 expression after IL-1β stimulation, both at 72 and 168 hrs, compared to CTR group (A,A1 and B,B1 respectively). Tempol (0.5mM and 1.0mM) significantly decreased these levels at both timepoints (A,A1 and B,B1). Data are representative of at least three independent experiments. One-way ANOVA test. *** $p < 0.001$ vs. CTR; ## $p < 0.01$ vs. IL-1β; ### $p < 0.001$ vs. IL-1β.

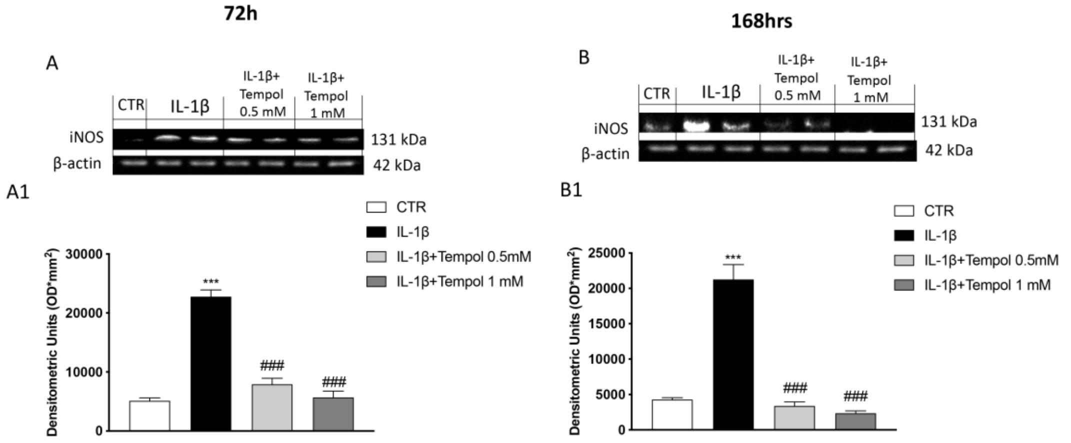

Figure 3S

Figure S3. Effects of tempol on iNOS expression. Western blot analysis of iNOS showed a significant increase in CC stimulated with IL-1β after 72 and 168 hrs (A,A1 and B,B1. Treatment with tempol (0.5mM and 1.0mM) considerably reduced iNOS expression already after 72 hrs and even more after 168 hrs (A,A1 and B,B1). Data are representative of at least three independent experiments. One-way ANOVA test. *** $p < 0.001$ vs. CTR; ### $p < 0.001$ vs. IL-1β.
